# Possible Biochemical Processes Underlying the Positive Health Effects of Plant-Based Diets—A Narrative Review

**DOI:** 10.3390/nu13082593

**Published:** 2021-07-28

**Authors:** Zoltan Szabo, Viktor Koczka, Tamas Marosvolgyi, Eva Szabo, Eszter Frank, Eva Polyak, Kata Fekete, Attila Erdelyi, Zsofia Verzar, Maria Figler

**Affiliations:** 1 Institute of Nutritional Sciences and Dietetics, Faculty of Health Sciences, University of Pecs, 7621 Pecs, Hungary; eszter.frank@etk.pte.hu (E.F.); eva.polyak@etk.pte.hu (E.P.); verzar.zsofia@pte.hu (Z.V.); maria.figler@aok.pte.hu (M.F.); 2 Department of Biochemistry and Medical Chemistry, Medical School, University of Pecs, 7624 Pecs, Hungary; koczka.viktor@pte.hu (V.K.); szabo.eva.dr@pte.hu (E.S.); 3 Doctoral School of Health Sciences, Faculty of Health Sciences, University of Pecs, 7621 Pecs, Hungary; 4 Institute of Bioanalysis, Medical School, University of Pecs, 7624 Pecs, Hungary; marosvolgyi.tamas@pte.hu; 5 Szentagothai Research Center, University of Pecs, 7624 Pecs, Hungary; 6 Institute for Translational Medicine, Medical School, University of Pecs, 7624 Pecs, Hungary; katae.fekete@gmail.com; 7 Institute of Health Insurance, Faculty of Health Sciences, University of Pecs, 7621 Pecs, Hungary; erdelyi.attila.if@gmail.com; 8 2nd Department of Internal Medicine and Nephrology Centre, Clinical Centre, University of Pecs, 7624 Pecs, Hungary

**Keywords:** vegan, plant-based diet, antioxidant, lipotoxicity, TMAO, IGF-1, mTOR, Neu5Gc, endotoxemia

## Abstract

Plant-based diets are becoming more popular for many reasons, and epidemiological as well as clinical data also suggest that a well-balanced vegan diet can be adopted for the prevention, and in some cases, in the treatment of many diseases. In this narrative review, we provide an overview of the relationships between these diets and various conditions and their potential biochemical background. As whole plant foods are very rich in food-derived antioxidants and other phytochemicals, they have many positive physiological effects on different aspects of health. In the background of the beneficial health effects, several biochemical processes could stand, including the reduced formation of trimethylamine oxide (TMAO) or decreased serum insulin-like growth factor 1 (IGF-1) levels and altered signaling pathways such as mechanistic target of rapamycin (mTOR). In addition, the composition of plant-based diets may play a role in preventing lipotoxicity, avoiding N-glycolylneuraminic acid (Neu5Gc), and reducing foodborne endotoxin intake. In this article, we attempt to draw attention to the growing knowledge about these diets and provide starting points for further research.

## 1. Introduction

The definition of plant-based diets is widely used, and its main focus is consuming raw or minimally processed vegetables, fruits, whole grains, legumes, nuts and seeds, spices, and herbs. Besides that, these diets often minimize or exclude all animal products [1]. A well-balanced plant-based diet is useful [2,3] for the primary prevention of several health conditions. It can be also used as a complementary treatment in chronic diseases, amongst others in cardiovascular diseases [2,4,5,6,7], obesity [8,9], certain types of cancer [10,11,12], type 2 diabetes mellitus (T2DM) [13,14,15], and stroke [16,17].

Several well-known healthcare institutes such as the Academy of Nutrition and Dietetics [18], the American Diabetes Association [19], the British Dietetic Association [20], the American Dietetic Association and Dietitians of Canada [21], the Directorate-General of Health of Portugal [22], and the German Nutrition Society [23] have given attention to plant-based nutrition, but these recommendations and position papers focus mainly on the macro- and micronutrient content of these diets and their potentially beneficial health roles. Although these diets are acceptable for example in the management of T2DM, with the evidence level B [19] and these recommendations mentioned above list a number of chronic diseases in which a vegetarian, vegan, or other plant-based diet might be beneficial, little is said about the physiological and biochemical mechanisms underlying these positive effects.

Plant-based diets could be an acceptable solution for improving and maintaining health as well as reversing some diseases, but it would also be important to know the underlying processes for recommendations. Therefore, the aim of this review is to provide an overview of some possible biochemical processes behind the positive physiological effects of these diets demonstrated in previous scientific literature.

## 2. Antioxidants

A typical Western-type diet is rich in refined carbohydrates (sugar), and saturated fats of animal origin, but low in fiber; however, high consumption of the first two ingredients can lead to a pro-oxidative state after each meal due to their active oxidation and simultaneous reactive oxygen species (ROS) formation [24]. Increasing evidence shows that this postprandial pro-oxidative state can induce inflammation after meals and is an important contributing factor besides obesity in several other chronic diseases, such as atherosclerosis, high systolic blood pressure, and insulin resistance [24,25]. This type of low-grade inflammation can lead to endothelial dysfunction; reduced insulin sensitivity and, consequently, reduced nitric oxide (NO) synthesis; and elevated oxidized low-density lipoprotein (LDL). Most of animal-based foods have significant proinflammatory and endothelial-dysfunction-inducing effects, although the exact mechanisms are not well understood [26]. These alterations can be detected even several hours after the consumption of animal-based foods. These factors can play a significant role in the development and progression of many diseases, such as metabolic syndrome, atherosclerosis, T2DM, hypertension, and stroke [24,27].

The acute negative effect of lipemia caused by a single high fat meal seems to be insignificant regard to compensatory mechanisms, which counteract most of these negative effects, but it is not negligible. Eventually, the undesirable effects accumulate and contribute to increased cardiovascular mortality [28]. Thus, consuming a variety of plant foods containing many antioxidants after every meal could be a reasonable way to reduce pro-oxidative state [29] caused by high calorie or high lipid containing meals.

The human metabolism can mitigate the negative consequences of this imbalance in many ways (superoxide dismutase, glutathione peroxidase, catalase, etc.). Numerous studies have shown that the use of exogenous antioxidants derived from plant foods can improve the efficiency of the endogenous enzymatic defense [30,31]. Plant-based foods contain the highest amounts of food antioxidants; by contrast, the antioxidant contents of animal-based foods are negligible, based on data for 3100 investigated foods, beverages, spices, herbs, and supplements used worldwide [32]. The consumption of plant foods containing a high number of antioxidants seems to be protective against the development of certain diseases (cancer, cardiovascular diseases, T2DM, osteoporosis, and neurodegenerative diseases) [33]. By contrast, animal-based foods contain certain catalytic molecules (e.g., iron, myoglobin, and hemoglobin), which, if consumed on a regular basis, could increase lipid peroxidation in the stomach. In different types of in vitro gastric model tests, 100-fold increase in the level of lipid hydroperoxides has been observed [34]. Proteins and lipids oxidized by these catalytic molecules, which can be found in significant quantities in processed meat, butter, and some heated plant-based oils (such as olive oil), may affect the development of Parkinson’s disease [35], inflammatory bowel disease [36], and insulin resistance [37].

Vitamin C, a reducing agent and electron-donor antioxidant, cannot be synthesized endogenously through the human metabolism, so the amount of ascorbic acid in the body depends on the dietary intake [38]. Vitamin C participates in the first line of antioxidant defense and is effective against the superoxide radical ion, hydrogen peroxide, hydroxyl radical, and singlet oxygen, as well as nitrogen oxide species. An in vitro experiment showed that vitamin C could increase the efficiency of vitamin E by decreasing tocopheroxyl radicals [39]. An eight-week-long interventional human study showed that the consumption of red fruit juice rich in anthocyanin and vitamin C increased the activity of superoxide dismutase [40]. Adequate vitamin C intake contributes to cardiovascular health, such as by improving blood pressure [41] and endothelial function [42].

Vitamin E is a plant-derived, potent peroxyl-radical-scavenging antioxidant. It can prevent the propagation of all free radicals in lipoproteins and membranes [43]. Animal and human studies have determined that vitamin E plays a major role in protecting the cerebellum from free radical species [44,45,46]. Four weeks of the daily consumption of roasted almonds caused a significant increase in plasma α-tocopherol concentrations but also a significant decrease in the level of IL-10, as well as lowering the inflammatory markers CAM-1, IL-1ß, and IL-6 in the serum [47].

Carotenoids are fat-soluble pigments that can be found in many plant-based foods. The antioxidant effect of carotenoids has been proven to be linked to their chemical structure. The presence of conjugated double bonds enables these molecules to accept electrons from reactive species and neutralize free radicals [48].

β-carotene is a provitamin that operates as a chain-breaking antioxidant but is less potent in the scavenging of peroxyl radicals. Zeaxanthin can effectively participate in the prevention of lipid peroxidation. A cross-sectional study showed that higher intake of fruits and vegetables rich in vitamin A (orange, peach, collard greens and kale) was associated with lower risk of developing glaucoma among older women. There was also a trend in risk reduction for β-carotene, lutein, and zeaxanthin, but it was not significant [49]. Lutein may play a protective role in reducing oxidative stress and damage of retina and optic nerve [50]. Lycopene is one of the most effective carotenoids in quenching singlet oxygen and participates in scavenging hypochlorous acid [51]. Lycopene is the predominant carotenoid in tomato, and it was shown that its bioavailability from tomato paste is higher than from fresh tomatoes [52]. In a randomized controlled study, the consumption of lycopene-rich tomato paste had protective effects against skin burn caused by UV light. The dietary intervention decreased the formation of erythema and inhibited the expression of matrix metalloproteinase-1 (MMP1) caused by UV radiation, which is a key regulator in the photoaging process [53].

A randomized controlled trial demonstrated that higher vegetable and fruit intake can increase the antioxidant property of HDL (high-density lipoprotein) due to the natural lycopene content of those foods [54]. In addition, it can reduce the risk of cardiovascular disease by decreasing the hydrogen-peroxide-induced oxidative injury of endothelial cells [55].

Due to the high antioxidant capacity of carotenoids, antioxidant and anticarcinogenic effects were hypothesized. To examine these phenomena, in two separate interventional studies, patients were supplemented with β-carotene. The β-Carotene and Retinol Efficacy Trial (CARET) demonstrated that the daily supplementation of β-carotene and vitamin A increased the prevalence of lung cancer in frequent smokers or in patients with substantial occupational exposure to asbestos in the USA [56]. These results were highly consistent with those found for β-carotene and vitamin E supplementation in the Alpha-Tocopherol Beta-Carotene (ATBC) cancer prevention study in male smokers in Finland [57]. The results of the long-term follow-up of the ATBC study confirm that β-carotene supplementation increases the lung cancer risk of smokers, regardless of the nicotine or tar content of cigarettes [58]. The best strategy for decreasing the prevalence of lung cancer among smokers is for them to quit smoking. Besides that, fruit and vegetable consumption have a protective effect in lung cancer’s etiology [59,60,61]. The dietary intake of carotenoids has some additional positive health effects, such as reducing the risk of T2DM [62] and metabolic syndrome [63].

There are plenty of phytochemicals of plant origin known to have potent biological antioxidant effects. The number of polyphenol-type antioxidant molecules alone could be more than 8000, as demonstrated in a previous study [33]. These compounds have significant positive effects (cancer, cardiovascular disease, neurodegeneration) on the human body in many ways [64]; however, their exact mechanisms of action are not yet fully understood. By manipulating many molecular signaling mechanisms such as PI3K, Akt, NF-κB, p53, and many others, these molecules positively affect the body’s oxidoreductive homeostasis [65].

The antioxidant effects of phenol-type compounds of plant origin are diverse. The phenol-type antioxidant activity depends on the compound’s ability to donate electrons or hydrogen, from which its potential for action as an antioxidant can be predicted. For instance, phenol-type molecules from fruits are capable of neutralizing superoxide anions, singlet oxygen, and lipid peroxides (‘scavenger function’) [66]. In addition, some flavonoids can recycle or reduce the body’s own antioxidant molecules and can also form chelates with metallic ions (Fe^2+^, Fe^3+^, and Cu^2+^), meaning that these molecules inhibit the pro-oxidative effect of metallic compounds. These chelates have an additional antioxidant effect similar to that of superoxide dismutase. This can serve as a further explanation for the positive physiological effects of antioxidant compounds [67,68,69].

Flavonoid–lipid and flavonoid–protein interactions are possible even if these compounds are present in small quantities. These interactions can induce numerous biological mechanisms, such as activating the body’s own antioxidant enzyme defense system and suppressing reactive oxygen species (ROS)-producing processes. Flavonoids and other antioxidant molecules of plant origin can contribute to a decreased absorption of lipid hydroxyperoxides. This may provide a further explanation for the positive physiological effects of antioxidant compounds [70,71].

Antioxidant supplementation, especially in the form of isolated components, seems to be ineffective [72,73]. Additionally, no beneficial effect on mortality has been described. In some cases, the use of antioxidant supplements may lead to undesirable health consequences, such as a higher occurrence of cancer and T2DM [74].

Data from clinical trials suggest that supplementation with an isolated component (vitamin E) or mixture of antioxidants (vitamin A and zinc) has unfavorable effects, such as increasing the incidence of hemorrhagic stroke and total mortality [75,76]. According to some researchers, antioxidant mixtures derived from natural sources are better than simple antioxidant formulas that is due to synergism between antioxidants [77].

Therefore, it seems beneficial to consume these antioxidants in their natural form as part of whole-plant foods. Fruits and vegetables are rich sources of polyphenols and, therefore, can have anti-inflammatory and antioxidant effects and could play a key role in the prevention or adjunctive therapy of different chronic diseases. Pomegranates are an excellent source of phytochemicals, such as anthocyanins, ellagic acid, and ellagitannins. In a randomized controlled trial, the consumption of one serving of pomegranate in its natural form decreased the plasma concentration of a potent inflammatory cytokine. This suppression of MCP1 (monocyte chemoattractant protein-1) was not detected after the ingestion of a dietary supplement rich in ellagic acid [78]. As antioxidants, polyphenol-containing fruits (e.g., strawberries) [28] and vegetables (e.g.,: kale) [79] can decrease LDL oxidation and as a result they can decrease the risk of coronary heart disease. Through different biochemical mechanisms (inhibiting regulatory enzymes and transcription factors involved in inflammation as well as scavenging free radicals) a diet high in antioxidants and polyphenols can prevent asthma, decrease the frequency of its exacerbation [80] and might be protective in the development and severity of different food allergies like peanut allergy [81].

Dietary antioxidants also have anti-aging effects due to their antioxidant and free radical scavenging potential. It is a well-known phenomenon that vegans, especially those who consume whole-food plant-based diet seems to look healthier and younger. Although this phenomenon has not been disclosed yet, the consumption of fruits and vegetables is a promising strategy to help maintaining youthful appearing of the skin [82,83]. Aging affects not only our skin, but other tissues also, for example our brain. In a follow-up study, women who had a higher intake of leafy greens or cruciferous vegetables from their diet showed the lowest degrees of cognitive decline [84].

Consuming various types of plant foods together can result in a difference between the expected and the measured antioxidant effects in favor of the latter. This phenomenon is often referred as “synergy”, which marks the additive positive effects of foodborne antioxidant molecules. [85]. Many examples prove the supremacy of consuming whole-plant foods over their isolated constituents [86], which gives us further verification that whole-plant consumption could be more beneficial most of the cases, due to their wide-range known and (probably still) unidentified micronutrient composition. However, to understand these complex relationships, the evaluation of each macro-, micronutrients, and phytochemicals should be examined in an extended way including the above mentioned “synergistic” phenomenon [87,88].

**Figure 1:**
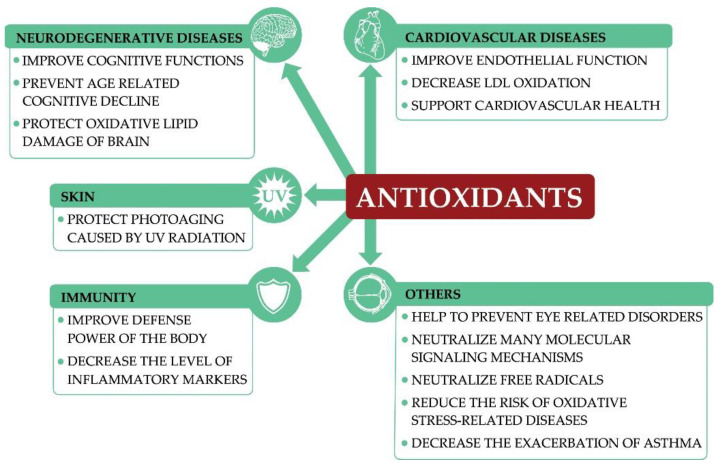
Influence of natural-source antioxidants on health.

From these few examples, it seems clear that these unique whole-plant food compounds can help to maintain overall state of health and even contribute to the prevention of certain diseases (Figure 1) through various mechanisms, which still have to be confirmed.

## 3. Lipotoxicity

Lipids have key importance, not only in the mechanism of action of antioxidants. Lipids may play a prominent role in the positive health effects of plant-based diets [89].

The accumulation of ectopic lipid in non-fatty tissues is called lipotoxicity, which is a complex condition caused by increased plasma free fatty acids reaching toxic levels in non-adipose tissues when fat cells’ normal fat-storing capability is compromised [90].

The presence of excess fatty acids leads to the accumulation of intramyocellular lipid (IMCL—lipid deposition within myocytes) species such as diacylglycerol (DAG), ceramide, and long-chain acyl-CoAs [91]. If these metabolites occur in ß-cells, they can disrupt their functions [92,93]. Healthy subjects with normal body weight and without diabetes were examined using nuclear MRI spectroscopy, and it was found that IMCL could be a good predictor of susceptibility to insulin resistance [94]. Other clinical studies suggest that vegans have significantly lower IMCL levels than omnivores [95,96]. Furthermore, plant-based diets have protective effects on ß-cell function by increasing glucose sensitivity, decreasing basal insulin secretion and the mean glucose level [97]. Applying a low-fat, plant-based diet leads to a decrease in free fatty acid levels and better glycemic control compared to a low-carbohydrate omnivorous diet [98].

The further consequences of lipotoxicity can include the induction of proinflammatory processes [99], oxidative stress [100], and mitochondrial dysfunction [101].

**Figure 2:**
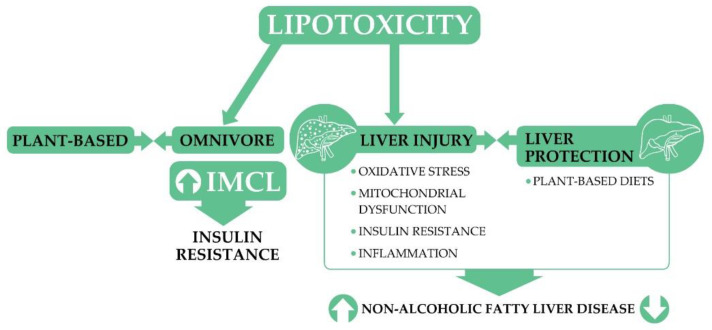
The potential role and consequence of plant-based diets in preventing lipotoxicity. IMCL: intramyocellular lipid.

Insulin resistance, mitochondrial dysfunction, and the alteration of intracellular signaling pathways lead to liver injury, and this can contribute to non-alcoholic fatty liver disease (NAFLD) [102]. The incidence of NAFLD is now at an endemic level, and it is currently the most common form of chronic liver disease worldwide, affecting about 25% of the general population [102,103]. The typical Western dietary pattern (high intakes of fast food, red meats, processed meats, full-fat dairy products, fried potatoes, high carbohydrate containing refined foods and soft drinks) is closely associated with the development and progression of NAFLD [104]. The connection between NAFLD and lipotoxicity is complex. Among the possible factors playing a role in the pathogenesis of NAFLD, free fatty acids seem to contribute to the development of lipotoxicity inducing lipid accumulation and lipotoxicity in liver cell cultures [105]. The connection between free fatty acid intake and insulin resistance has been reported in several studies [106,107,108,109]. The mitochondrial dysfunction caused by palmitic acid-induced oxidative stress can increase the damaging effects of ROS and disrupt insulin signaling [110]. The high dietary fat intake characteristic for Western-type diets can increase the serum free fatty acid levels, which is an independent risk factor for the development and worsening of NAFLD [111]. The possible consequences of lipotoxicity are summarized in Figure 2.

Adherence to plant-based diets appears to be protective against NAFLD (mainly because enhanced glycemic control, improved insulin sensitivity, and decreased chronic inflammation) [112,113], but further clinical trials are needed in order to clarify this relationship in more detail. A better understanding of lipid irregularity may eventually modify the concept of lipotoxicity as a key pathogenic factor in many diseases [102,114].

## 4. Trimethylamine N-Oxide

The mechanisms discussed in the previous sections can also have profound effects on the cardiovascular system through a number of mechanisms. More recently, studies have also focused on the trimethylamine N-oxide (TMAO) molecule, which reveals a highly significant relationship between diet and gastrointestinal and cardiovascular health.

TMAO is an amine oxide with the formula (CH_3_)_3_NO; it is an oxidized form of trimethylamine (TMA). TMAO is primarily formed from nutritional substrates from the metabolism of carnitine, dimethylglycine, phosphatidylcholine, choline, and betaine by intestinal microflora in the colon [115]. These substrates are mainly derived from products of animal origin but may also be of plant origin. Choline- and carnitine-rich foods include animal-based foods such as eggs, dairy, harslet, red meat, poultry, seafood, and fish [116], but carnitine can be found only in limited amounts in plant foods [117,118]; for example, ground beef contains about 400 times more carnitine than whole wheat bread does [119]. TMA may be present in the diet, but its dietary intake is negligible. After the precursors have been transformed into TMA by bacteria, it is absorbed into the bloodstream. TMA can be transformed into TMAO by hepatic enzymes called flavin monooxygenases (FMO1 and FMO3). Unabsorbed TMA is decomposed into methylamine, dimethylamine (DMA), and ammonia within the colon [120].

The biologically active stereoisomer form of carnitine (L-carnitine) participates in fatty acid metabolism, the maintenance of plant and animal cell homeostasis, and signaling pathways in both plants and animals. L-carnitine, derived from the diet or supplements, is absorbed by active and passive transport through intestinal cell membranes. The liver and the kidneys are the main organs responsible for the biosynthesis of carnitine. Carnitine plays an essential role in the transport of long-chain fatty acids into the mitochondria, which is a rate-limiting step in fatty acid oxidation [121]. L-carnitine is more bioavailable for vegetarians, and their daily loss is also minimal due to their adaption to low-carnitine diets compared to those following omnivorous diets [122].

The precursor for TMA production in vegetarians, vegans, and omnivores is phosphatidylcholine, which is the main dietary source of choline. Soy, cruciferous vegetables such as cauliflower, Brussels sprouts, cabbage, etc., and whole grains are plant-based sources of choline. Although Brussels sprouts are the most abundant source of choline among plant foods, after its consumption instead of an increase, a significant decrease in the urinary TMAO profile has been observed. This can be due to two indole-containing compounds that are potent inhibitors of human FMO3 [123]. This study raises the possibility that plant-based choline sources could have different effect on TMAO production compared to animal-based sources due to their phytochemical composition.

### TMAO and Its Clinical Importance

According to prospective observational studies, plasma TMAO levels are related to the incidence of cardiovascular diseases. Both in vivo studies in mice and in vitro studies on human cells have suggested that physiological levels of TMAO stimulate the expression of inflammatory cytokines and adhesion molecules [124]. TMAO can induce the formation of foam cells, and it was also found that the formation of foam cells triggered by oxidized LDL was enhanced by TMAO [125,126]. Moreover, clinical studies in patients with heart failure [127,128] and hemodialysis [129] proposed a preatherogenic role for TMAO in the development of atherosclerosis.

Numerous studies suggest that human gut microbiota may be a double-edged sword. Lifestyle and dietary choices can have a beneficial or detrimental effect on the human health by altering the gut microbiota [130,131]. It plays a role in the development of cardiovascular diseases due to its production of TMAO from carnitine and choline. Red meat consumption has been found to be one of the main risk factors for the development of cardiovascular diseases [132]. Researchers have shown that red meat caused the highest TMAO concentration in the blood compared to the concentrations observed in white meat eaters and non-carnivorous (vegetarian) groups [133]. In 2019, a study was carried out in which omnivores and vegans/vegetarians consumed 450 mg/day of choline for about two months. The results showed that the TMAO levels were elevated from the baseline in both groups, although the vegans/vegetarians had much lower TMAO and platelet aggregation than the omnivores. However, as time passed, the microbiomes of the vegans/vegetarians started to adapt to the choline supplementation, which resulted in higher concentrations of TMAO and platelet aggregation by the end of the study. Despite these differences, at the end of the study’s two-month period, the vegans/vegetarians showed elevated but still much lower levels of TMAO than the omnivorous group [134]. Supplementation of TMAO’s substrates may alter TMAO levels in any dietary groups, even those who primarily had non (or less)-TMAO producing gut flora.

Omnivorous individuals ingest 2–12 μmol/kg of carnitine of body weight/day, which provides 75% of the body’s carnitine sources [117,121]. As carnitine is mainly present in animal-based foods, vegetarians and vegans only consume very small amounts of carnitine in their diets (around 1 μmol/kg/day). Therefore, those following plant-based diets obtain more than 90% of their carnitine through biosynthesis [121].

Previous research has reported the cardioprotective effects of plant-based diets [2,4,5,6,7], which may be partially explained by vegetarians’/vegans’ reduced capacity to produce TMAO [135]. A recent study of healthy subjects concluded that TMAO production from carnitine is higher in omnivores than vegans [136]. In this context, the modification of the gut microbiota composition and diversity with plant-based diets appears to be a useful option in the treatment of diseases related to high TMAO levels [137]. In addition, several studies have proven that the decreased TMAO levels can be partially explained by the remodeling potential of plant compounds, which leads to decreased TMA formation. In an animal study, resveratrol-supplemented chow feed altered the gut microbiome in mice, which might have contributed to the decreased gut microbial TMA production [138]. These results could also be corroborated in a randomized placebo-controlled clinical trial in healthy subjects supplemented with resveratrol-containing grape pomace extract. After four weeks supplementation, the TMAO levels were significantly decreased in the experimental group suggesting microbiota remodeling in the gut [139].

Carnitine supplementation in a regular diet can increase TMAO production [140].

In a short-term randomized controlled trial, fecal microbial transplantation from donors following a vegan diet changed the composition of the gut microbiota but did not affect the TMAO production in obese, omnivorous, atherosclerotic patients. Nonetheless, the study had the limitation that the participants insisted on following their own omnivorous diets after this intervention [141].

The level of TMAO production can vary from person to person. Many studies have mentioned that individuals with different phenotypes presumably produce different amounts of TMAO in the gut [136]. The clinical relevance of this claim has not yet been proven, so further research is needed to find out more about the mechanisms of action involved.

Therefore, the findings of these studies show that the gut microbiota may play an important role in plant-based dietary interventions aiming to decrease the risk of developing cardiovascular diseases. Further clinical studies are needed to establish the beneficial effects of plant-based diets, especially for decreasing the level of TMAO production in the gut.

## 5. Insulin-like Growth Factor-1

In the previous sections, we examined components consumed through the diet or metabolized in the body from dietary components, but we now want to discuss different regulatory mechanisms that are highly influenced by dietary factors.

Human insulin-like growth factor-1 (IGF-1), or somatomedin C, is an anabolic hormone produced by hepatocytes that consists of 70 amino acids in a single polypeptide chain, with intramolecular disulfide bridges. The insulin-like growth factor-1 system includes IGF-1, IGF-1 receptor (IGF-1R), and IGF-binding proteins (IGFBPs) [142].

IGF-1 regulates many functions of cell metabolism, mediates the growth effect of human growth hormone (hGH), promotes the proliferation of many cells, and inhibits apoptosis; as a consequence, it could promote the survival of malignant cells [143,144].

IGF-1’s serum levels progressively decline with age [145] and are influenced by nutrition [146]. The effects of certain nutrients on serum IGF-1 and its expression at the mRNA and protein levels seem to be decisive; in different tissues such as the liver and intestines, the levels of IGF-1 tend to decrease with fasting and are restored with refeeding [147]. Beyond energy intake from different macronutrients, a higher intake of protein seems to regulate the level of IGF-1, which may contribute to a higher body mass in early childhood [148,149,150].

Significant energy and protein restriction (especially restricting the essential amino acids [151]) can reduce IGF-1 plasma levels in both animals and humans in general [152]. The growth hormones IGF-1 and IGFBPs play a role in the growth of children by stimulating the longitudinal growth of the bones [148]. In vegan children, a low and limited intake of essential amino acids and proteins can affect their growth velocity [153]. Recent data suggest that there are no additional nutritional risks among vegan children compared to omnivores [154].

In theory, following high-calorie, low nutrient-dense diets (such as the typical Western-type diet) early in life adversely program the principal components of metabolic syndrome and other conditions by promoting growth acceleration [155]. In contrast, in some earlier studies, relative undernutrition and slower growth (compared to Western-type diet) in early life may result in a lower risk of developing cardiovascular diseases later [156]. The early life environment as well as early nutrition (both undernutrition [157] and overnutrition [158]) can play a key role in the later health and development of different diseases (better known as “Developmental Origins of Health and Disease” (DoHaD) theory [159], or early programming theory [160]), but the complex biochemical processes behind this phenomenon (and the potential role of IGF-1) needs to be clarified. Therefore, optimal early nutrition, with lower calorie and higher nutrient-content compared to Western-type intake, can play a key role in the prevention of developing chronic diseases (e.g., obesity, cardiovascular diseases) in adulthood.

### IGF-1 and Cancer

Cancer is one of the leading causes of death globally, and the incidence of cases increased by 33% between 2007 and 2017 [161,162,163].

Data on molecular mechanisms suggest that the activation of different genes combined with IGF-1 signaling can perturb the normal homeostasis of the cell, and these irregularities can be found in different carcinogenic processes [164,165,166,167]. High levels of circulating IGF-1 and IGFBP-3 in the blood are related to a higher risk of certain cancers, such as colorectal, prostate, and breast cancers [168,169,170].

Higher levels of energy and protein intake [171], as well as milk [172,173,174] and meat [175] consumption, are associated with elevated IGF-1 levels, which may increase the risk of developing prostate cancer in men [176,177,178] and breast cancer in women [171,179,180]. By contrast, the consumption of plant-based diets (especially vegan diets with a low consumption of soy milk) can reduce IGF-1 levels [181]. Moreover, both epidemiological studies [179,180] and clinical data [182] have shown that following a plant-based diet (the regular consumption of fruit, vegetables, legumes, or whole grains) may reduce the risk of developing certain cancerous diseases, such as colorectal, prostate, and breast cancer [11,18,183,184,185]. Based on data from the Adventist Health Study 2, which is the study with the largest cohort of vegans and vegetarians to date, the tumor risk was significantly lower in vegans/vegetarians than in non-vegetarians. In both sexes, vegan diets showed a remarkably higher protection against cancerous diseases. The authors explained these differences as being due to, among other factors, the differences in IGF-1 levels [12].

It is not well understood what mediates the positive effect of plant-based diets on the IGF-1 system (plant-based protein, high fruit and vegetable consumption, a high fiber intake, and low fat consumption); further evidence and clinical research are needed. IGF-1 should be targeted for cancer prevention, and the regulation of its level could be a potential therapeutic point in general and lifestyle medicine [186].

## 6. Mechanistic Target of Rapamycin

In close association with IGF-1, there is another potent regulatory mechanism that is highly influenced by dietary factors: the mechanistic target of rapamycin (mTOR).

mTOR is a serine/threonine protein kinase from the phosphatidylinositol-3-kinase (PI3K) superfamily. It is present in all eukaryotes and plays a key role in regulating cell growth and proliferation, cellular energy levels, oxygen levels, and mitogenic signals [187]. mTOR has two functional units (mTOR complex 1 (mTORC1) and mTOR complex 2 (mTORC2)), which act as central connectors of nutrient signaling pathways and are involved in the regulation of the cell cycle [188]. It was discovered in the 1970s as a result of the search for the target of rapamycin, a macrolide unit produced by *Streptomyces hygroscopicus* bacteria. Rapamycin is a selective inhibitor of mTOR. Rapamycin inhibits the transcriptional activity of cytokines by suspending their production, but it also has antifungal and antitumor effects, and it is used as an immunosuppressant [189]. Rapamycin appears to have life-extending properties [190,191], but its widespread recreational use has been inhibited by its side-effect profile (hyperlipidemia, high blood sugar, anemia, and inflammation of the oral mucosa) [192]. mTOR could be a key factor in the development and progression of many conditions (such as metabolic diseases, obesity, cancer, and ageing). The manipulation of mTOR mechanisms through dietary interventions seems to be suitable for the prevention and treatment of these conditions.

### 6.1. mTOR Complex 1

mTORC1 controls major regulatory processes (as a nutrient sensor, responding to dynamic changes in amino acid levels, ATP, and growth factor signaling), apoptosis, and stress responses [191,193,194]. These inputs are capable of synergizing and antagonizing each other, enabling the cell to fine-tune the action of mTORC1. Therefore, the deregulation of mTORC1 activation is associated with many diseases (including cancer and T2DM).

Amino acids such as leucine, arginine, and glutamine are important signals for mTORC1 activation [193,195,196,197]. Moreover, leucine-mediated mTORC1–ribosomal protein S6 kinase beta-1 (S6K1) signaling induces insulin resistance by the phosphorylation of insulin receptor substrate 1 (IRS-1). Leucine-mediated mTORC1–S6K1 signaling also plays an essential role in adipogenesis, thus increasing the risk of obesity-related insulin resistance [194].

In normal conditions, mTORC1 regulates lipid accumulation in fat cells, primarily through storage as white adipose tissue (WAT). The adipocyte-specific deletion of raptor (which is an mTOR binding partner that is necessary for mTOR signal transduction, binding to mTORC1, and the phosphorylation of mTOR-catalyzed substrates) reduces the amount of WAT in adipose-specific raptor knockout mice and increases the oxidation of fatty acids [198]. Accordingly, long-term chronic mTORC1 hyperactivity causes increased lipogenesis in the liver and WAT, which can lead to obesity and insulin resistance [199].

### 6.2. mTOR Complex 2

mTORC2 plays a key role in cell survival and the regulation of anabolic processes [200,201]; the endogenous activity of mTORC2 is localized to the plasma membrane and mitochondrial and endosomal spaces with distinct sensitivities to phosphoinositide 3-kinase (PI3K) and growth factor signaling, promoting Akt phosphorylation by encouraging Akt to localize to the plasma membrane. The stimulation of cells by insulin promotes the S473 phosphorylation of Akt by mTORC2. In addition, mTORC2 phosphorylates the AGC kinase family members and activates Akt, serum- and glucocorticoid-induced protein kinase (SGK), and protein kinase C (PKC), which regulates cell survival, cell cycle progression, and anabolism [199,202]

mTORC1 and mTORC2 cooperate closely, regulating many different processes (autophagy, apoptosis, neurodevelopment, cell migration, dendritic arborization, and adipocyte formation). Although, mTOR pathways are very diverse in the eukaryotic cells most details of their regulated processes are still unclear [202]. Whether plant-based diets can act via mTOR signaling pathway leading better health and decreasing the risk of chronic diseases needs to be further investigated in human trials.

#### 6.2.1. PI3K–mTOR Pathway in Cancer

The PI3K–Akt–mTOR pathway plays an important role in the regulation of multiple cellular functions, affecting both anabolism and catabolism [194]. The dysregulation of the PI3K–Akt–mTOR pathway is involved in the development of numerous human diseases, such as cancer [203]. mTOR overactivity is mainly caused by the activation of the PI3K–Akt signaling pathway and occurs in virtually every type of tumor cell, including breast cancer cells, colorectal cancer cells, and gastric cancer cells [204]. In the case of breast cancer, the overactivation of this pathway leads to protein synthesis, which, in turn, contributes to the increase in tumor cell proliferation and cell growth. Moreover, increased mTOR signaling can stimulate angiogenesis and may confer resistance to estrogen endocrine therapy [205].

The PI3K–Akt–mTOR pathway is also a strong regulator of autophagy [206] and is involved in the development and promotion of pathological disorders such as cancer. Activated mTORC1 inhibits autophagy through the inhibitory phosphorylation of Unc-51, such as by autophagy activating kinase (ULK1) [193]. Thus, hampering PI3K-Akt-mTOR-mediated autophagy may be an important therapeutic strategy in the treatment of various tumors. The activation of 5′ AMP-activated protein kinase (AMPK) can downregulate ULK1 and, therefore, inhibiting both early and late phase of autophagy [207].

#### 6.2.2. Caloric Restriction, Fasting, and mTOR

The cell senses nutrient supply through different signal transductional pathways (AMPK, recombination-activating gene (Rag), PI3K/Akt, etc.). Different states of nutrition alter the cell’s energetic homeostasis through various pathways (anabolism and catabolism). Amino acids, especially leucine, are capable of inhibiting AMPK, which leads to mTORC1 activation [194]. For this reason, restricting protein and calories can be an option for suppressing mTOR [208]. Short and longer time fasting upregulated in both in vitro cell and in vivo animal study the expression of farnesyl-diphosphate farnesyltransferase 1 (FDFT1), which acts as a critical tumor suppressor in colorectal cancer by negatively regulating Akt–mTOR–hypoxia inducible factor-1α (HIF1α) signaling and, therefore, resulted in slower tumor growth [209].

Animal models and clinical studies showed a wide range of beneficial effect of longer-time caloric restriction for several health conditions (e.g., obesity, T2DM, cardiovascular disease, cancers, neurologic disorder), which appears to be safe if it is conducted appropriately (e.g., intermittent fasting) [210].

### 6.3. Plant-Based Diets and mTOR

Energy restriction has been recognized as a life-extending factor [211,212,213]. In many cases, the application of energy restriction is impossible or difficult to achieve. The effect of restricting protein consumption on longevity, can be similar to that of energy restriction [214]. In this case, the inhibition of both the IGF-1 and mTOR signaling pathways, as described earlier, may be responsible for an increased life expectancy [215]. Otherwise, a fully plant-based (“vegan”) diet has the possibility to enhance longevity through protein and amino acid restriction and other key mechanisms (fibroblast growth factor 21 (FGF21) induction, gut microbiome diversity), but it has to be confirmed [216].

The quantity and quality of protein consumption can alter mTOR’s activity. As discussed earlier, amino acids (of which leucine has the strongest effect) are important signals for mTORC1 activation [217]. Therefore, a significant reduction in leucine intake reduces the activity of the mTORC1 signaling pathway as much as a reduction in total amino acid intake [218,219] that can be an effective tool for the prevention of several chronic diseases like T2DM, obesity, and cancer [197]. As animal-based foods (meat, eggs, milk, and dairy products) contain the highest levels of leucine, the only way to reduce leucine intake is to eliminate or at least strictly reduce the consumption of these food sources and increase the consumption of plant-derived foods that are low in leucine [194].

Based on different in vitro cell culture data whole-plant food components have inhibitory effect on mTORC1 activation [220]. Furthermore, polyphenols, flavonoids, and curcumin are regarded as natural inhibitors of mTORC1 and exert antidiabetic and anti-obesity effects [221,222,223,224,225]. There is evidence that polyphenol supplementation can effectively reduce fasting blood glucose levels in both T2DM patients or individuals who are at risk of developing diabetes [226]. Anti-obesity effect of different phytochemicals seems promising based on in vitro and animal studies, but evidence for clinical relevance is very low because of the limited number of clinical trials [227].The synergistic effects of these phytonutrients could be much greater than the effects of consuming each component alone or only the active ingredients of whole foods in isolation [228], but more clinical trials are needed that can corroborate this theory.

Despite the fact that there are very limited clinical data available demonstrating the exact relationship between plant-based diets and mTOR, it seems plausible that further studies would confirm the theoretical link between mTOR complexes and dietary factors.

## 7. Other Factors

### 7.1. N-Glycolylneuraminic Acid

By consuming animal flesh and meat products, certain molecules that can be found in farm animals but cannot be produced by the human body itself may trigger immune responses and chronic inflammation. This molecular mechanism could be another explanatory factor in the relationship between the consumption of animal-based foods and certain pathological processes.

Sialic acids are well-known molecules that were discussed by numerous research groups in the 1940 and the 1950s [229,230]. Sialic acid and its derivatives are believed to play an important role in immunological processes [231]. The two most common forms of sialic acid in mammals are N-acetylneuraminic acid (Neu5Ac) and N-glycolylneuraminic acid (Neu5Gc) [232]. Human cells cannot synthesize Neu5Gc because of the irreversible mutation of the cytidine monophospho-N-acetylneuraminic acid hydroxylase (CMAH) gene [233]; however, small amounts can be found in different human cells due to dietary intake [234]. Since plants, fungi, and the microorganisms in our bodies are unable to produce Neu5Gc, the primary source of the Neu5Gc molecule in the human body is animal-based foods [233,235] with the highest concentrations in caviar (446–531 μg/g), beef (134–231 μg/g), and lamb (19–57 μg/g) [236].

Neu5Gc exposure causes the production of anti-Neu5Gc antibodies, which can participate in the propagation of autoimmune processes. In addition, most cancerous cells exhibit a high affinity for the accumulation of the Neu5Gc molecule, resulting in the production of anti-Neu5Gc antibodies. Subsequently, the already-mentioned “low-grade inflammation” condition develops, contributing to the survival, further proliferation, and angiogenesis of malignant cells [237]. These mechanisms may also contribute to the association of red meat consumption with the prevalence of T2DM [236].

**Figure 3:**
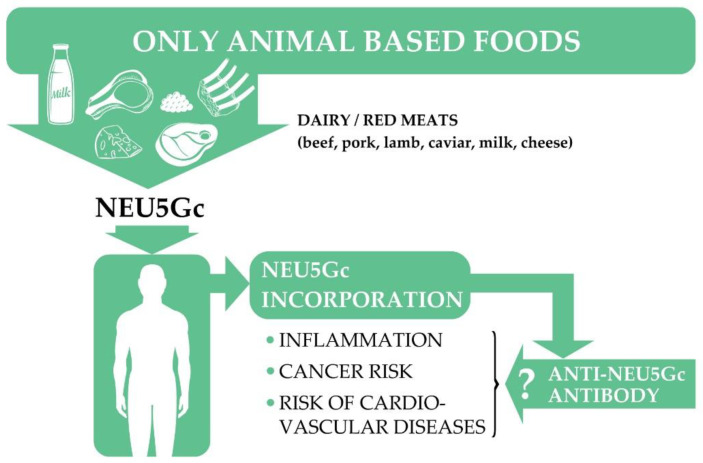
The potential role of animal food-derived Neu5Gc in the risk of certain diseases. Neu5Gc: N-glycolylneuraminic acid.

The Neu5Gc molecule is also highly accumulated in epithelial cells, especially at atherosclerotic sites [233]. Thus, it is suggested that anti-Neu5Gc antibodies can contribute to the progression of atherosclerosis [238]. The potential role of Neu5Gc in the development of anti-Neu5Gc antibody-related diseases is summarized in Figure 3.

Intriguingly, SARS-CoV-2 may use host Neu5Gc molecules, similar to MERS-CoV. A current hypothesis suggests that SARS-CoV-2 has a sialylated glycan shield, which may lend support to the idea that Neu5Gc and its derivatives are possible virus receptors [239].

Although the data described here may be of interest, anti-Neu5Gc-antibody-related disorders need to be confirmed by well-designed human clinical trials [240].

### 7.2. Endotoxemia

Endotoxins (Lipopolysaccharides (LPS)) are structural components of Gram-negative bacteria and therefore the main targets of antibody production [241] and possible contributors of inflammation [242]. When the LPS level in the blood is elevated, the production of toll-like receptor-2 (TLR2) and toll-like receptor-4 (TLR4) can be stimulated [226]. As TLR4 is one of the activators of the NF-κB protein complex, its inadequate regulation may be related to inflammatory processes when NF-κB stimulates the transcription of proinflammatory genes [227].

In the Bruneck study, LPS was found in healthy individuals in low concentrations (median 14.3 pg/mL), but at levels of 50 pg/mL or above, they increased the risk of atherosclerosis, that was more pronounced in ex-smokers and current smokers [109,243]. Dietary factors, as high saturated fat diet can also increase postprandial plasma levels of LPS leading to inflammatory response [244].

Based on human epidemiological studies, consumption of several prebiotics (e.g.,: insoluble dietary fiber, resistant dextrin, galacto-oligosaccharides, oligofructose, inulin) that is typical in plant-based diets can decrease the level of LPS [245], while many foodstuffs typical for Western-diet (pork, turkey, soft cheese, ice cream, and chocolate) contained TLR2 stimulants in an in vitro study, and, therefore, can induce endotoxemic state [246].

As we mentioned in relation to TMAO, the gut flora have the ability to significantly modify these processes also [247]. The mechanisms underlying the connection between nutrition, microbiome, health, and certain diseases go far beyond the scope of this narrative review, but the use of plant-based diet in this context also seems beneficial [248,249].

Contradictory data are available concerning the relation of on the one hand certain diets and on the other hand endotoxemia and postprandial inflammation [250,251], but it seems that consuming foods rich in saturated fats as well as several food typical for Western-type diet can increase the levels of LPS for a short time, even in healthy subjects [252], while different dietary factors typical for plant-based diets can decrease LPS levels in a few weeks on average [245]. To confirm the relative usefulness of plant-based diets in this context need to be confirmed with more well-designed clinical trials.

## 8. Summary

The adoption of plant-based diets is becoming popular in the Western world, so it is necessary for practicing healthcare professionals to be well informed about these diets. Support for the implementation of plant-based diets by dietitians will be more effective if they better understand the positive physiological consequences of these diets. While this review is not comprehensive, we have tried to describe some of the huge amount of scientific evidence confirming the efficacy and usefulness of plant-based diets. Thus, explanatory mechanisms could become partly recognizable too. Nevertheless, the exact mechanisms involved in a wide range of positive health effects remain unclear. This review draws attention to the need for further evidence-based, high-quality studies.
